# *Burkholderia thailandensis* strain E555 is a surrogate for the investigation of *Burkholderia pseudomallei* replication and survival in macrophages

**DOI:** 10.1186/s12866-019-1469-8

**Published:** 2019-05-15

**Authors:** A. Kovacs-Simon, C. M. Hemsley, A. E. Scott, J. L. Prior, R. W. Titball

**Affiliations:** 1 College of Life and Environmental Sciences, University of Exeter, Geoffrey Pope Building, Stocker Road, Exeter, EX4 4QD UK; 2 CBR Division, Defence Science and Technology Laboratory, Porton Down, Salisbury, SP4 0JQ UK

**Keywords:** *Burkholderia pseudomallei*, *Burkholderia thailandensis* strain E555, Transcriptome, Proteome, Virulence

## Abstract

**Background:**

*Burkholderia pseudomallei* is a human pathogen causing severe infections in tropical and subtropical regions and is classified as a bio-threat agent. *B. thailandensis* strain E264 has been proposed as less pathogenic surrogate for understanding the interactions of *B. pseudomallei* with host cells.

**Results:**

We show that, unlike *B. thailandensis* strain E264, the pattern of growth of *B. thailandensis* strain E555 in macrophages is similar to that of *B. pseudomallei*. We have genome sequenced *B. thailandensis* strain E555 and using the annotated sequence identified genes and proteins up-regulated during infection. Changes in gene expression identified more of the known *B. pseudomallei* virulence factors than changes in protein levels and used together we identified 16% of the currently known *B. pseudomallei* virulence factors. These findings demonstrate the utility of *B. thailandensis* strain E555 to study virulence of *B. pseudomallei*.

**Conclusions:**

A weakness of studies using *B. thailandensis* as a surrogate for *B. pseudomallei* is that the strains used replicate at a slower rate in infected cells. We show that the pattern of growth of *B. thailandensis* strain E555 in macrophages closely mirrors that of *B. pseudomallei*. Using this infection model we have shown that virulence factors of *B. pseudomallei* can be identified as genes or proteins whose expression is elevated on the infection of macrophages. This finding confirms the utility of *B. thailandensis* strain E555 as a surrogate for *B. pseudomallei* and this strain should be used for future studies on virulence mechanisms.

**Electronic supplementary material:**

The online version of this article (10.1186/s12866-019-1469-8) contains supplementary material, which is available to authorized users.

## Background

The bacterial pathogen *Burkholderia pseudomallei* causes meliodosis, a severe disease of humans in tropical and subtropical regions [1–3]. The clinical manifestations of melioidosis range from an acute sepsis to chronic localised disease to latent infection, which can reactivate decades later from an as yet unknown tissue reservoir [4, 5]. Community-acquired disease is most likely a consequence of the bacterium in soil or water entering through cuts or skin abrasions. In small animal models of disease, the bacterium is much more infective by the airborne or intranasal routes [6, 7]. This might be consistent with the reported cases of disease in apparently healthy US helicopter crew during the Vietnam War, as a consequence of the inhalation of soil-derived dusts containing bacteria [8]. The bacterium is considered a bio-threat agent because of the high infectivity by the airborne route [2, 9]. A recent study has estimated that worldwide there are 165,000 human melioidosis cases and 89,000 deaths per year [1]. There is no licensed vaccine for the prevention of disease [10], and the bacterium is inherently resistant to many antibiotics [2, 11]. Against this background, there is an urgent need for medical countermeasures to the disease, and the development of new approaches to disease control will be dependent on an understanding of the mechanisms by which *B. pseudomallei* establishes infection. Whilst a range of studies have characterized some of the virulence factors of *B. pseudomallei* [2, 12–14], it is also clear that a much broader range of genes and proteins are implicated in playing a role in disease [13, 15, 16]. However, studies on virulence of the bacterium are necessarily constrained by the requirement to handle the bacterium under containment level 3 conditions.

*Burkholderia thailandensis* is a close relative of *B. pseudomallei* and is also found in the soil in tropical regions of the world [17]. The genomes of *B. pseudomallei* and *B. thailandensis* are very similar with two highly syntenic chromosomes, which have similar numbers of coding regions, similar assignments of encoded proteins to families and similar numbers of horizontally acquired genomic islands [18]. However, infections of humans with *B. thailandensis* are rare and the bacterium can be handled outside of a high containment laboratory. Several previous studies have used *B. thailandensis* as a surrogate for *B. pseudomallei*, to study virulence mechanisms [19–37]. These studies have used *B. thailandensis* isolates, such as strain E264, that replicate more slowly than *B. pseudomallei* in cell cultures [38, 39]. These strains lack the capsular polysaccharide of *B. pseudomallei*, which is a key virulence determinant [14, 40]. However, a minority of *B. thailandensis* strains possess a *B. pseudomallei*-like capsule [21, 40], though they are not virulent in murine models of disease [40]. In this study we set out to assess one of these strains as a surrogate for *B. pseudomallei*, in the hope that this would more faithfully mimic the behavior of *B. pseudomallei* in cell culture, and could be used to study virulence.

## Results

### Genome sequencing

We selected *B. thailandensis* strain E555 (a natural isolate previously described by Sim et al. [40]) for our study because, unlike most *B. thailandensis* strains, it possesses a capsular polysaccharide which is similar to *B. pseudomallei* [40, 41]. We sequenced the *B. thailandensis* strain E555 available in our laboratory. Sequence data are available at DDBJ/ENA/GenBank under the accession SJET00000000. In *B. pseudomallei,* the *wcb* operon, which includes capsular polysaccharide genes, consists of 20 genes (*wcbA*-*T* [42, 43]) of which 19 are also encoded in the *B. thailandensis* strain E555 genome (*wcbN* is not present) (Additional file 3: Table S1 C). In addition to the capsule, *B. thailandensis* strain E555 has been shown to exhibit several *B. pseudomallei*-like phenotypes such as colony wrinkling, resistance to human complement binding, and survival in macrophages [40]. However, despite these similarities, *B. thailandensis* strain E555 shows the same level of virulence in mice as other *B. thailandensis* strains [40]. Using RAST [44] we predicted 6508 open reading frames in our *B. thailandensis* strain E555 genome sequence data (ORFs; Additional file 3: Table S1 A and B). We compared the amino acid sequences of *B. pseudomallei* strain K96243 proteins to *B. thailandensis* strain E555 and *B. thailandensis* strain E264 and found 4535 proteins in both *B. thailandensis* strain E555 and strain E264, 94 only in *B. thailandensis* strain E555 and 380 only in *B. thailandensis* strain E264. The remaining 914 proteins were not found in either *B. thailandensis* strain and of these 233 were encoded by genes located in the genome islands in *B. pseudomallei*.

### Infection model

**Fig. 1:**
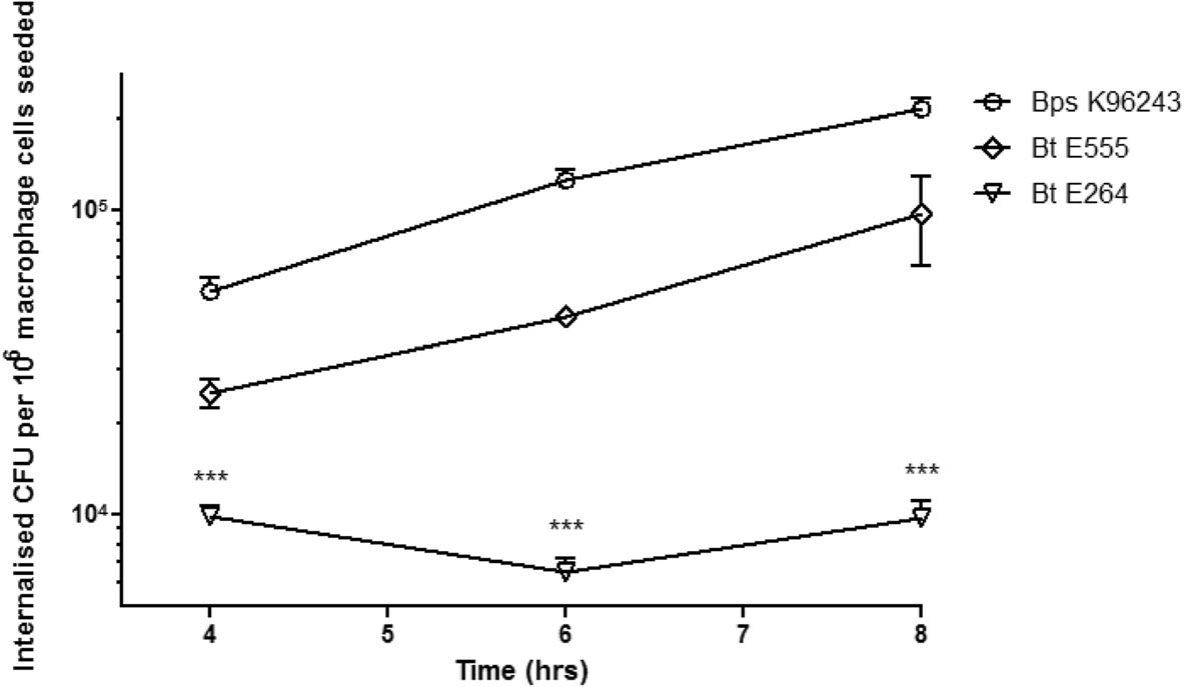
Intracellular survival of *Burkholderia* strains in mouse macrophages. J774A.1 mouse macrophages were exposed to *B. thailandensis* strain E555 at an MOI of 10 for 2 h. Following removal of the extracellular bacteria, infected macrophage cells were incubated with kanamycin (1 mg/ml in the first 2 h and 0.25 mg/ml afterwards) for 1, 2, 4 and 6 h and the mean numbers (with standard errors of the mean) of intracellular bacteria were determined. *B. thailandensis* strain E264 and *B. pseudomallei* strain K96243 values were adapted from Wand et al. [39]. There was no statistically significant difference between the growth rate of *B. thailandensis* strain E555 and *B. thailandensis* strain E264, or between the growth rate of *B. thailandensis* strain E555 and *B. pseudomallei* strain K96243 at any time point (*p* > 0.05). In contrast, the difference between the growth rate of *B. pseudomallei* strain K96243 and *B. thailandensis* strain E264 was significant: *p* < 0.001 at 4 h, 6 h and 8 h post-infection, (indicated by ***)

We first infected J774A.1 mouse macrophage cells with *B. thailandensis* strain E555 and the number of intracellular bacteria was measured at intervals. We compared this data with data previously obtained in our laboratory using an identical method with *B. pseudomallei* strain K96243 or *B. thailandensis* strain E264 which is commonly used as a surrogate for *B. pseudomallei* [39]. *B. thailandensis* strain E555 and *B. pseudomallei* strain K96243 had similar growth rates in macrophages (doubling times are 2.02 h for *B. pseudomallei* strain K96243 and 2.06 h for *B. thailandensis* strain E555 over the 4 h period between 4 h and 8 h post-infection), while the growth of *B. thailandensis* strain E264 was slower and showed a different growth pattern (*p* values between *B. pseudomallei* strain K96243 and *B. thailandensis* strain E264 were *p* < 0.001 at 4 h, 6 h and 8 h post-infection, see Fig. 1).

### Analysis of the global bacterial transcriptome

**Fig. 2:**
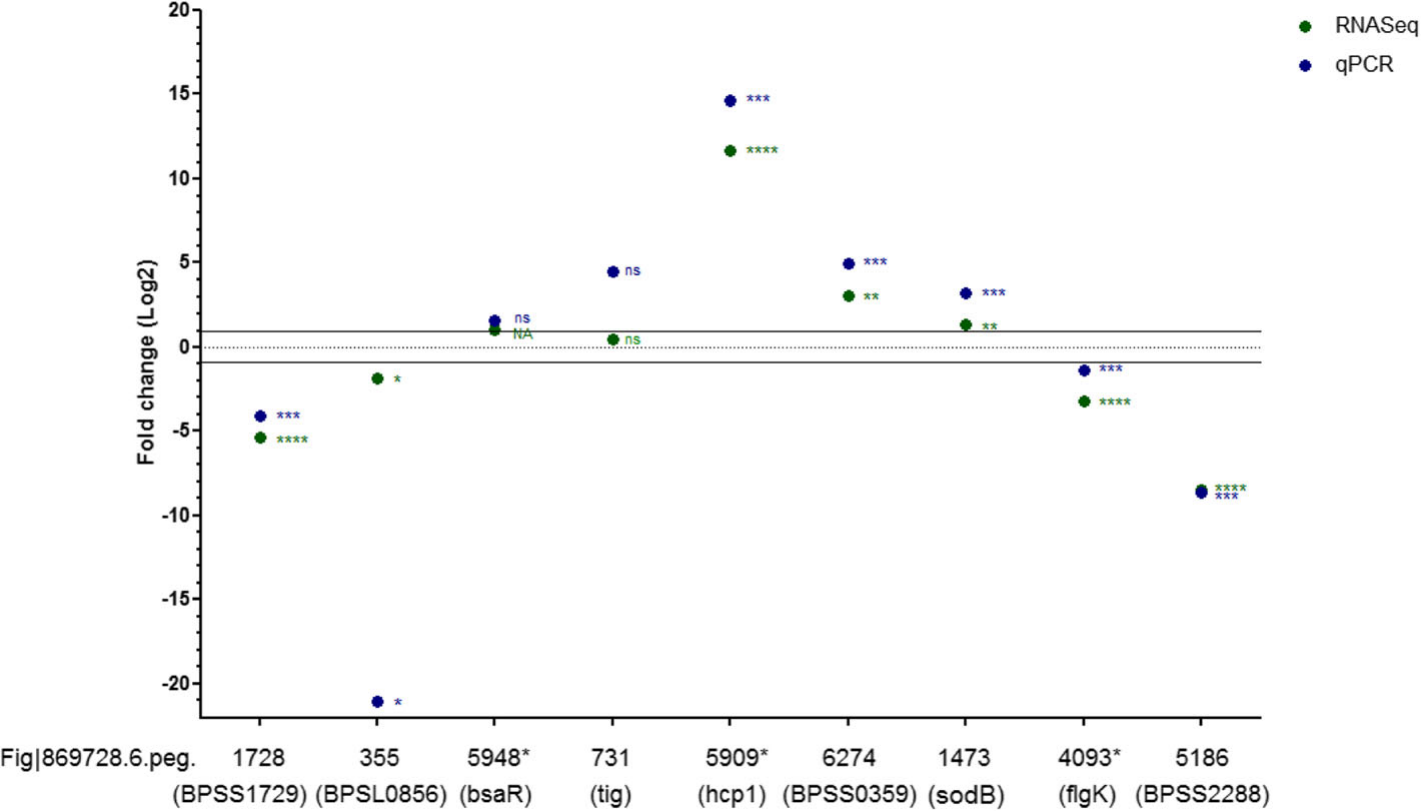
Validation of RNASeq data by RT-qPCR. RNASeq (green) and RT-qPCR (blue) data at 6 h post-infection in macrophages. Tested genes: [i] 3 genes (Fig|869,728.6.peg.5909*, 6274, 1473) with significantly increased, [ii] 4 genes (Fig|869,728.6.peg.4093*, 5186, 1728, 355) with significantly decreased and [iii] 2 genes (Fig|869,728.6.peg.5948*, 731) without significantly increased level of expression during macrophage infection (a known virulence factor* was included in each group). *B. pseudomallei* homologs of these genes are shown in brackets. Internal control: 23S rRNA. Expression data is detailed in Additional file 4: Table S2. Horizontal lines show 2-fold change in expression cut-off. Asterisks on the graph indicate statistical significance

To investigate how *B. thailandensis* strain E555 adapts to the intracellular environment in J774A.1 mouse macrophage cells, the transcriptional and translational landscapes of the bacteria were profiled, using RNA-seq or mass spectrometry, after overnight growth in broth culture and during macrophage infection. Nine genes (Additional file 4: Table S2) were selected to validate the data generated from the RNA-seq study using RT-qPCR (the 23S rRNA was used as an internal control) [45, 46] (Fig. 2).

**Fig. 3:**
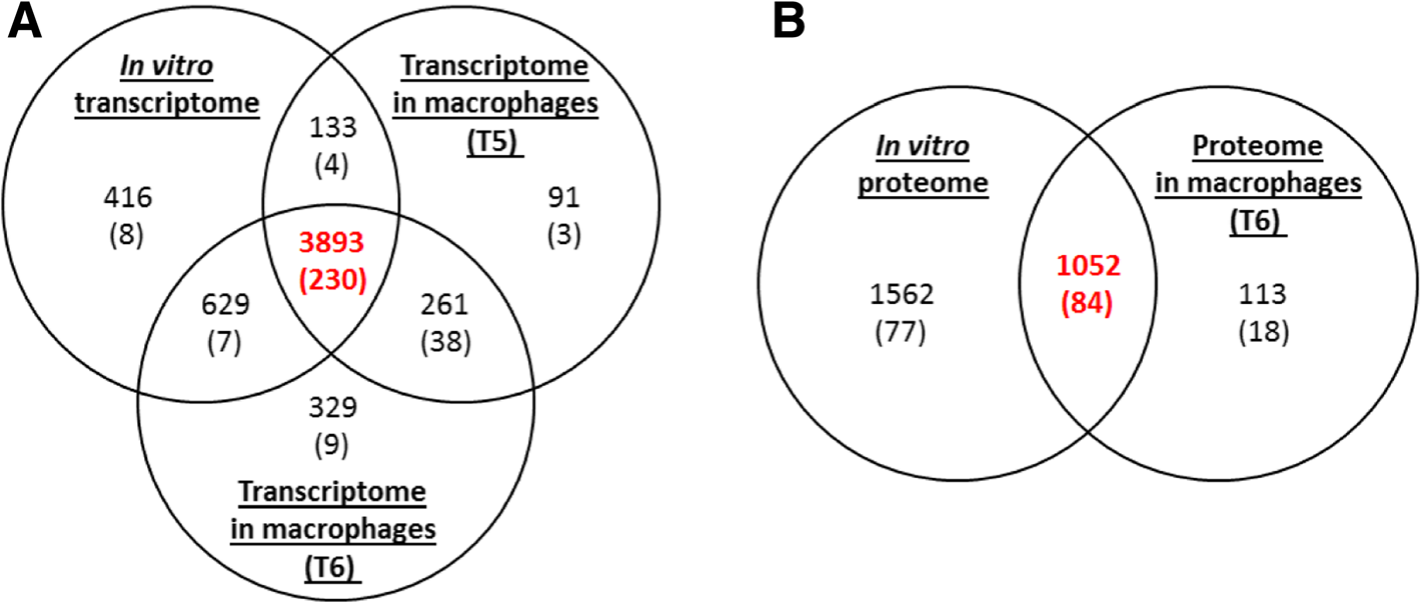
Venn diagram of the transcriptional and translational landscapes of *B. thailandensis* strain E555 grown in vitro in LB broth and in macrophages. **a** Transcriptome. **b** Proteome. Figures in brackets indicate numbers of known virulence factors

We identified broadly similar numbers of genes expressed in broth-cultured bacteria (*n* = 5071) or in bacteria from macrophages (*n* = 4378 and *n* = 5112 at 5 and 6 h post-infection) and most (*n* = 3893) were expressed in all 3 samples (Fig. 3 a and Additional file 5: Table S3). The abundance of transcripts at 5 h or 6 h post-infection (Fig. 3 a) showed a high correlation (R = 0.904; Additional file 1: Figure S1). Unique transcripts were more abundant at 6 h (*n* = 329) than at 5 h (*n* = 91) post-infection indicating that the adaptation of the bacteria to the intracellular environment was still in progress between 5 h and 6 h post-infection. Expression of all of the *wcb* genes (*n* = 19) encoded in the *B. thailandensis* strain E555 genome was detected in the transcriptomes of bacteria grown in broth and bacteria grown in macrophages (at 5 h and 6 h post-infection) (Additional file 7: Table S5.F). Overall, we concluded that the RNA-seq analysis provided a robust picture of the *B. thailandensis* transcriptome.

### Analysis of the global bacterial proteome

**Fig. 4:**
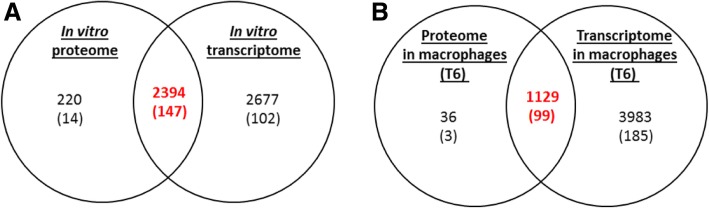
Correlation between the transcriptional and translational landscapes of *B. thailandensis* strain E555 grown in vitro in LB broth or in macrophages. Venn diagram of the transcriptome and the proteome of *B. thailandensis* strain E555 (**a**) during in vitro (broth) growth or (**b**) during in vivo (macrophage) growth. Figures in brackets indicate the numbers of known virulence factors

For proteomics, we used an immunomagnetic method to first purify bacterial cells from infected macrophages. The protocol was assessed by visualising the protein extracts separated by SDS-PAGE. We tested three different antibodies against lipopolysaccharide, capsular polysaccharide or against *B. pseudomallei* cells, each conjugated separately onto magnetic beads. Using antibodies against capsular polysaccharide, with an incubation time of 25 min at 4 °C, was the most efficient way of recovering bacteria from macrophage lysates (data not shown). Bacterial proteins were then identified by mass spectrometry (Additional file 6: Table S4). We detected lower numbers of proteins than corresponding gene transcripts. For over 90% of the proteins we identified the corresponding mRNA transcripts but conversely we could not detect the proteins corresponding to the majority of the transcripts (Fig. 4). These transcripts were not close to the limits of detection, suggesting that the inability to detect the corresponding protein was not linked to low levels of gene expression (Additional file 1: Figures S2 and S3). In contrast to the transcriptome data, not all of the proteins encoded by the *wcb* operon were found in the proteome; we detected 12 in broth grown bacteria and 7 in bacteria isolated from macrophages (Additional file 7: Table S5.F).

**Fig. 5:**
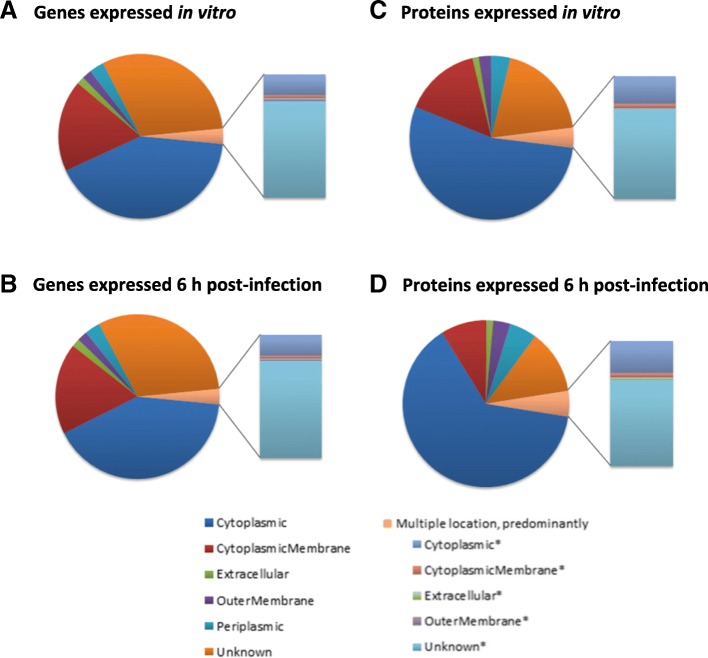
Distribution of the cellular localisation of *B. thailandensis* strain E555 transcripts and proteins detected in culture and in infected macrophages. **a** Bacterial transcripts detected during in vitro growth in culture. **b** Bacterial transcripts detected 6 h after infection of macrophages. **c** Bacterial proteins detected during in vitro growth in culture. **d** Bacterial proteins detected 6 h after infection of macrophages

Compared with transcriptome mapping, our proteomic analysis revealed the over-representation of cytoplasmic proteins. In contrast, proteins of unknown location were under-represented compared with transcriptome data sets (Fig. 5), possibly reflecting the limitations of pSORTb in predicting exported proteins [47], which would be difficult to detect in our proteome samples.

### Intracellular metabolism

We investigated which cellular processes are required and which are dispensable in bacteria during infection of macrophages. We found up-regulation, of several fatty acid biosynthetic enzymes both in the 6 h transcriptome and in the 6 h proteome. Genes encoding proteins with roles in the valine, leucine and isoleucine biosynthetic and degradation pathways were also up-regulated at 6 h post-infection. In addition, four (*argT*, *hisM*, *hisQ*, *hisP*) out of five components of a lysine, arginine, ornithine and histidine ABC-type transporter and a branched chain amino acid transporter (*livK*, *livH*, *livM*, *livG*, *livF*) were up-regulated 6 h after infection. We found down-regulation of a number of genes involved in nitrogen metabolism (metabolic enzymes and two-component systems). Linked to this pathway, glutamate metabolism was down-regulated. Finally our findings that cysteine and methionine metabolism were downregulated in bacteria isolated from macrophages suggests that these pathways are dispensable during infection.

### Comparison of the significantly regulated transcriptome and proteome

We next identified genes and proteins that were significantly (*p* < 0.05) up- or down-regulated at least two-fold in bacteria isolated from macrophages, compared with broth-cultured bacteria. We found that 11% of genes (265 up-regulated and 438 down-regulated) were differentially regulated at 5 h post-infection (Additional file 7: Table S5), and 15% of genes (396 up-regulated and 583 down-regulated) were differentially regulated at 6 h post-infection (Additional file 7: Table S5 and Additional file 1: Figure S4.A). A total of 242 genes were up-regulated at both 5 h and 6 h post-infection. 23 genes were up-regulated only at 5 h post-infection and 154 genes were up-regulated only at 6 h post-infection. Among the 23 genes, 6 were known virulence factors of which 5 are components of the T3SS-3. Despite the larger number of up-regulated genes, only an additional 10 genes are known virulence factors of the 154 genes specific for 6 h post-infection (Additional file 7: Table S5.D). For our subsequent studies we used transcript data at 6 h post-infection.

**Fig. 6:**
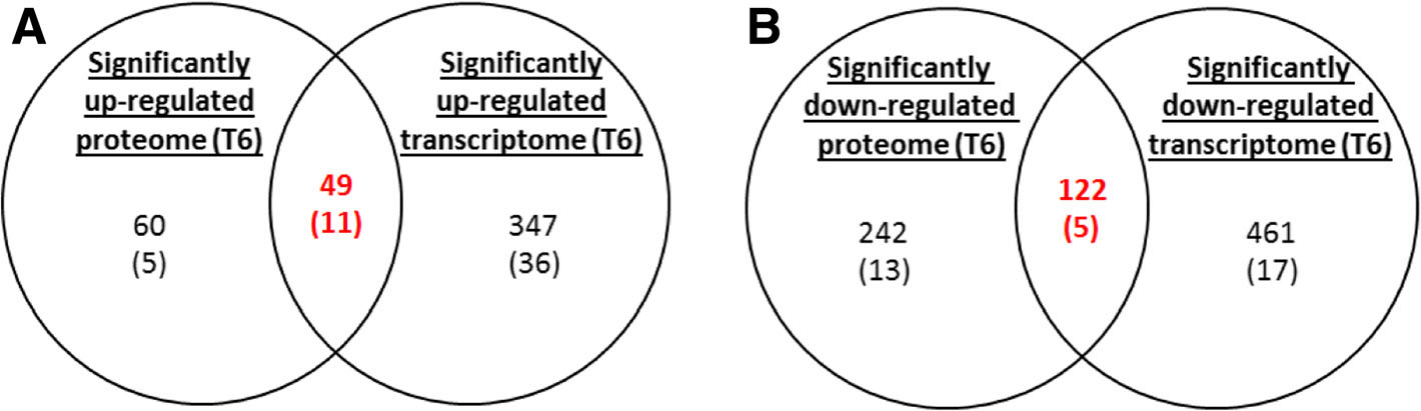
Comparison of the significantly regulated genes and proteins during macrophage infection. **a** Over-expressed genes and proteins. **b** Repressed genes and proteins. Figures in brackets indicate the numbers of known virulence factors

In parallel, the proteomic landscape was investigated. We applied the same statistical criteria as with the transcriptomic data and identified 109 up-regulated and 364 down-regulated proteins 6 h post-infection (Additional file 7: Table S5 and Additional file 1: Figure S4.B). The number of up- or down-regulated proteins was smaller than the number of up- or down-regulated genes (Fig. 6). At 6 h post-infection we found 45% overlap between significantly up-regulated proteins and genes and 34% overlap between significantly down-regulated proteins and genes (Fig. 6). We found one up-regulated protein but with decreased corresponding mRNA, and 16 genes with decreased protein levels but with increased corresponding mRNA levels (Additional file 7: Table S5).

**Fig. 7:**
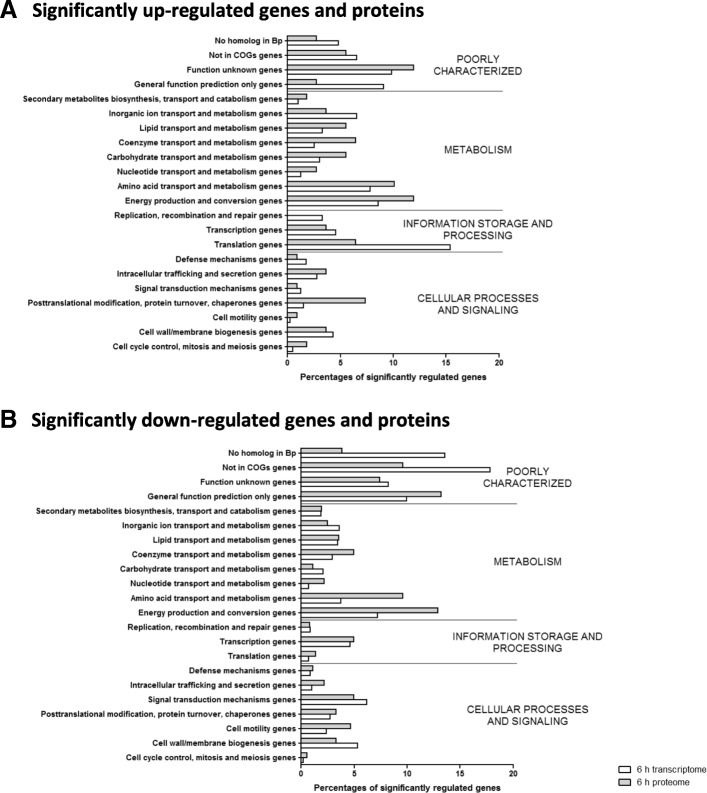
Functional classification by COG designation of *B. thailandensis* genes and proteins significantly regulated during macrophage infection. Bars indicate percentages of genes or proteins relative to the total number genes and proteins significantly up-regulated (**a**) or down-regulated (**b**)

The groups which showed the greatest degree of enrichment in bacteria isolated from macrophages were genes encoding proteins involved in translation, and proteins involved in post-translational modification functions (Fig. 6 a). Proteins associated with amino acid transport and metabolism, and energy production were over-represented in the significantly down-regulated proteome but not in the significantly down-regulated transcriptome (Fig. 7 b).

### Virulence factors

We compiled a list of 326 genes encoding virulence factors of *B. pseudomallei* which had been identified from previous experimental studies (Additional file 2: Supplementary References, Additional file 8: Table S6.A). Of these we found 267 in both *B. thailandensis* strain E555 and strain E264, 17 only in *B. thailandensis* strain E555 and 19 only in *B. thailandensis* strain E264. The remaining 23 virulence-associated factors were not found in either *B. thailandensis* strain (Additional file 8: Table S6). Of the 17 candidate virulence-associated factors found in both *B. thailandensis* strain E555 and *B. pseudomallei* but not in *B. thailandensis* strain E264, 9 are implicated in capsule biosynthesis, 6 genes encoded hypothetical proteins, 1 encoded a cell surface protein and 1 a non-ribosomal peptide synthetase. Of the 23 virulence-associated factors found only in *B. pseudomallei* strain K96243, 16 are hypothetical proteins or have either unknown or predicted function, 6 are predicted to be involved in transport, metabolism or catabolism of amino acids, lipids or secondary metabolites, and 1 is predicted to have a role in intracellular trafficking and secretion. These proteins might merit further attention as potential virulence determinants of *B. pseudomallei*. All or most of the genes in T6SS-1, T6SS-2, T6SS-3, T6SS-5 and T6SS-6 are present in the genome of the *B. thailandensis* strain E555 but the T6SS-4 locus is missing (only two genes, the homologs of BPSS0166 and BPSS0185, are present).

In bacteria isolated from macrophages at 6 h post-infection expression of 284 of the virulence associated genes and 102 of the virulence associated proteins were detected (Fig. 4 b). Most (97%) of the virulence-associated factors detected in the proteome were also detected in the transcriptome (Fig. 4 b). Of these, 47 genes and 16 proteins were up-regulated compared to broth grown bacteria (Fig. 6 a) and some virulence-associated factors were down-regulated (Fig. 6 b).

**Fig. 8:**
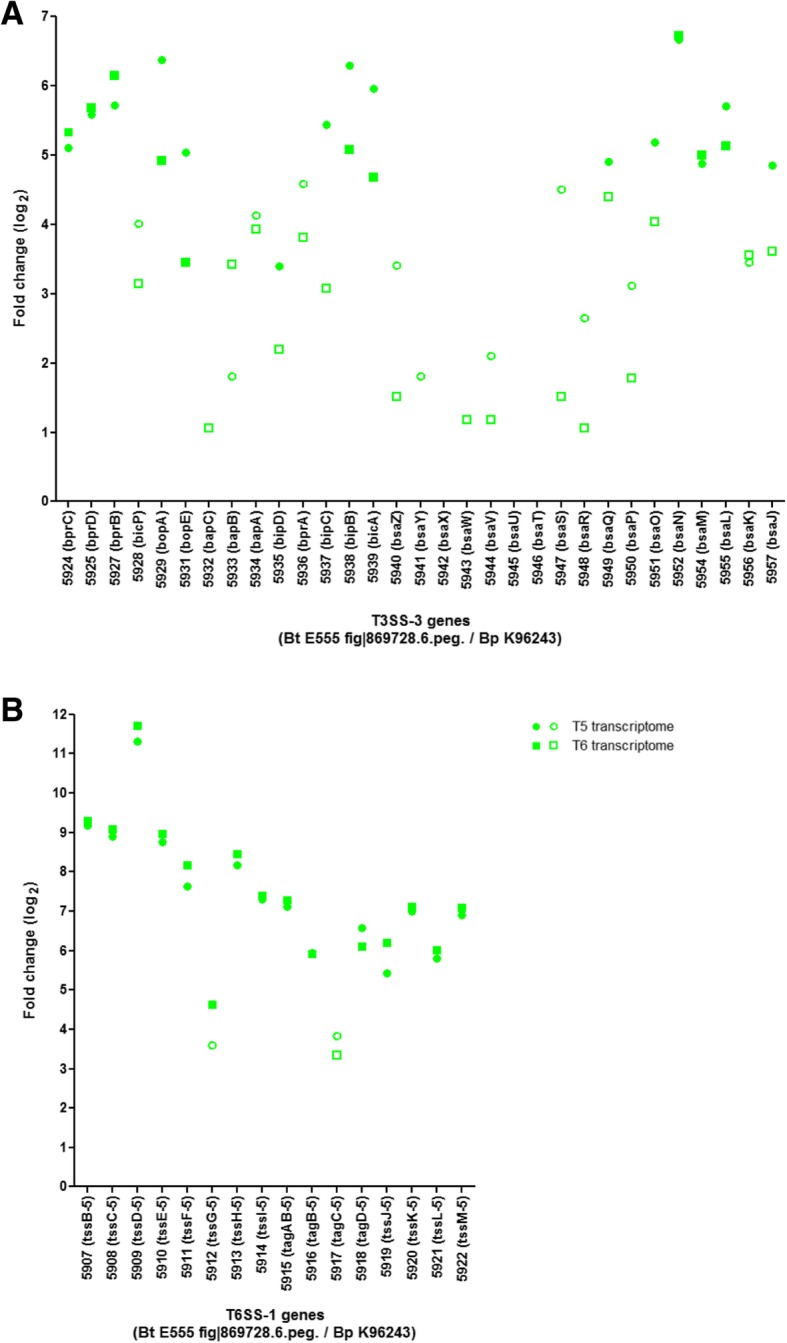
Expression of the T3SS and T6SS-1 genes in the bacterial transcriptome during infection of macrophages. Up-regulation of the (**a**) T3SS and the (**b**) T6SS-1 genes. Homologs of the T3SS and T6SS-1 genes in *B. pseudomallei* are shown in brackets. Green circles: data for 5 h post-infection, green rectangles: data for 6 h post-infection, filled shapes represent significant up-regulation. Data was taken from Additional file 7: Table S5

We found that 14 (56%) of the 25 most highly up-regulated genes in *B. thailandensis* isolated from macrophages are known virulence factors of *B. pseudomallei* (Table 1). 11 of these genes encode components of the type VI secretion system-1 (T6SS-1) (Fig. 8 b) and the remaining genes encode BsaN, BimA and BprB which are associated with virulence [13, 48–50]. We also found that 10 of the 30 type III secretion system-3 (T3SS-3) genes were upregulated at 6 h post-infection (Fig. 8 a, Additional file 5: Table S3.D and Additional file 7: Table S5). One of the T3SS-3 genes (*bsaN*) was in the 25 most up-regulated genes (Table 1). RT-PCR data validated the up-regulation of *bsaR*, a component of the T3SS-3, and *tssD-5* (*hcp1*), a component of the T6SS-1 (Fig. 2).

**Table 1:** List of 25 most up-regulated genes in the transcriptome of *B. thailandensis* strain E555 during infection of macrophages after 6 h post-infection

Bt E555 fig|869,728.6. peg.	log_2_ fold change in T6 transcriptome	Bt E555 gene function assigned by RAST	Bp Sanger ID	Bp gene name	Bp gene product	Significantly upregulated in T5 transcriptome?	Significantly upregulated in T6 proteome?
**5909**	11.7106	Hcp	BPSS1498	tssD-5 (hcp1)	type VI secretion system protein TssD-5	Yes	Yes
5896	9.5536	N-acetylmuramoyl-L-alanine amidase (EC 3.5.1.28)	BPSS1490	–	N-acetylmuramoyl-L-alanine amidase	Yes	Yes
**5907**	9.3004	Uncharacterized protein ImpB	BPSS1496	tssB-5	type VI secretion system protein TssB-5	Yes	Yes
**5908**	9.0842	Uncharacterized protein ImpC	BPSS1497	tssC-5	type VI secretion system protein TssC-5	Yes	Yes
**5910**	8.9729	Uncharacterized protein similar to VCA0109	BPSS1499	tssE-5	type VI secretion system protein TssE-5	Yes	
5897	8.5387	Plasmid pIB6 ORFA DNA	BPSS1491	–	hypothetical protein	Yes	Yes
2756	8.4671	TonB-dependent hemin, ferrichrome receptor	BPSS0244	–	BhuR (Outer membrane heme receptor)	Yes	No
**5913**	8.4574	ClpB protein	BPSS1502	tssH-5	type VI secretion system protein TssH-5	Yes	No
**5911**	8.1660	Protein ImpG/VasA	BPSS1500	tssF-5	type VI secretion system protein TssF-5	Yes	No
3093	8.0481	SyrP-like protein	BPSL1785	–	hypothetical protein	Yes	Yes
3100	7.8577	ABC-type siderophore export system, fused ATPase and permease components	BPSL1779	–	siderophore biosynthesis related ABC transport protein	Yes	No
3113	7.4295	Non-ribosomal peptide synthetase modules, pyoverdine	BPSL1778	–	siderophore related no-ribosomal peptide synthase	No	No
**5914**	7.3994	VgrG protein	BPSS1503	tssI-5	type VI secretion protein TssI-5	Yes	No
**5915**	7.2906	hypothetical protein	BPSS1504	tagAB-5	type VI secretion system-associated protein TagAB-5	Yes	Yes
5839	7.2027	Fig. 00464790: hypothetical protein	BPSL3352	–	hypothetical protein	Yes	
**5920**	7.1177	Uncharacterized protein ImpJ/VasE	BPSS1509	tssK-5	type VI secretion system protein TssK-5	Yes	Yes
**5922**	7.0900	IcmF-related protein	BPSS1511	tssM-5	type VI secretion system protein TssM-5	Yes	Yes
6277	7.0886	Hemin uptake protein	BPSS0362	–	hypothetical protein	Yes	No
3095	6.9269	Ferrichrome transport system permease protein FhuB (TC 3.A.1.14.3)	BPSL1783	–	iron-hydroxamate transporter permease subunit	No	No
**5952**	6.7155	Type III secretion thermoregulatory protein (LcrF,VirF,transcription regulation of virulence plasmid)	BPSS1546	bsaN	regulator of type III secretion system effector proteins BsaN (T3SS-3)	Yes	No
5900	6.4369	Fig. 00456189: hypothetical protein	no homolog			Yes	No
**5899**	6.4344	Autotransporter adhesin	BPSS1492	bimA	Burkholderia intracellular motility A, BimA	Yes	Yes
**5919**	6.1933	putative lipoprotein	BPSS1508	tssJ-5	type VI secretion system protein TssJ-5	Yes	Yes
3092	6.1826	hypothetical MbtH-like protein, PA2412 homolog	BPSL1786	–	hypothetical protein	Yes	No
**5927**	6.1495	DNA-binding response regulator	BPSS1522	bprB	two-component response regulator	Yes	No

Known virulence factors of *B. pseudomallei* are shown in bold in the first column

Four genes involved in iron acquisition were up-regulated in *B. thailandensis* strain E555 isolated from macrophages (Table 1; Bt E555 fig|869,728.6.peg.2756, 3100, 6277 and 3095; in *B. pseudomallei* BPSS0244, BPSL1779, BPSS0362 and BPSL1783, respectively) highlighting the importance of iron acquisition during infection.

The known importance of lipopolysaccharide [51] during infection by *B. pseudomallei* was mirrored by our finding of increased expression of the *rfbA*-*rfbB* lipopolysaccharide ABC-transporter genes and increased expression of lipopolysaccharide biosynthetic genes and proteins in *B. thailandensis* strain E555 within macrophages. We also found up-regulation of *B. thailandensis* strain E555 fig|869,728.6.peg.5896 (BPSS1490 in *B. pseudomallei*) which encodes a peptidoglycan biosynthetic enzyme (Table 1). Of the capsule genes only *wcbA* and *wcbK* were significantly up-regulated (in the 6 h transcriptome and in the 6 h proteome, respectively).

The role of the SyrP-like protein Bt E555 fig|869,728.6.peg.3093 (BPSL1785 in *B. pseudomallei*) in virulence has not been tested experimentally in *B. pseudomallei* but there is evidence that it plays a role in the virulence of *Pseudomonas syringae* through the control of syringomycin production [52] and this gene was highly up-regulated in *B. thailandensis* strain E555 isolated from macrophages (Table 1).

Using our data we investigated whether up-regulated genes or up-regulated proteins were more likely to be virulence-associated factors. Significantly up-regulated genes identified 16% of the known virulence-associated factors, whilst significantly up-regulated proteins revealed only 5% (Table 2 and Additional file 9: Table S7). However, the proteome analysis identified virulence factors with higher degree of confidence than the transcriptome analysis (14.7% versus 11.9%). Combining the significantly up-regulated gene and protein datasets did not significantly improve the predictive power. But looking at the genes common in both the significantly up-regulated transcriptome and the significantly up-regulated proteome increased the likelihood of detection of virulence factors (22%; Table 2). In comparison, the likelihood of randomly identifying a virulence-associated factor from the genome is approximately 5%.

**Table 2:** Summary of bacterial virulence factors detected in the transcriptome and in the proteome during infection of macrophages

Groups	Description	Total number of genes	Number of virulence factors	Virulence factors in	
				proportion of the total known virulence factors in *B. thailandensis* E555 (302)	proportion of the genes detected in the group (column 3)
A	Significantly up-regulated in the transcriptome	396	47	15.6%	11.9%
B	Significantly up-regulated in the proteome	109	16	5.3%	14.7%
A + B	All genes in Groups A and B	456	52	17.2%	11.4%
A = B	Common genes in Groups A and B	49	11	3.6%	22.4%

See the lists of genes in each group in the Additional file 9: Table S7

Some proteins, such as those which are membrane located or secreted, are difficult to recover while the corresponding transcripts are not subject to these constraints. Since the most likely cellular locations of virulence factors are at the cell surface and in the extracellular milieu, this might limit the utility of proteomics for the identification of virulence factors. However, extracellular, outer membrane and periplasmic proteins were found in similar proportions in the proteomes and in the corresponding transcriptomes. Nevertheless, all proteins are initially translated in the cytoplasm allowing their detection in this cellular compartment. Therefore, proteome analysis might also provide confident data for identification of classic virulence candidates, especially because the majority of cellular processes occur at protein level and not at transcript level.

## Discussion

Central to this study is the proposition that *B. thailandensis* is a surrogate for understanding the interactions of *B. pseudomallei* with host cells. Most previous studies have used *B. thailandensis* strain E264 for these studies, but our work reveals that unlike *B. thailandensis* strain E264, *B. thailandensis* strain E555 grows at similar rate to *B. pseudomallei* in macrophages. A previous report showed that a *B. pseudomallei* mutant lacking capsular polysaccharide showed no difference in intracellular growth rate compared to the wild type [53]. Therefore the similar behaviour of *B. thailandensis* strain E555 to *B. pseudomallei* in host cells is unlikely to be due simply to the presence of the similar capsule in these different bacteria. Consistent with this we find that the overall genetic makeup of *B. thailandensis* strain E555 is more similar to that of *B. pseudomallei* strain K96243 than to the overall genetic makeup of *B. thailandensis* strain E264.

In this study we also compared the relative utilities of transcriptomic or proteomic approaches to identify genes which are differentially regulated after infection. Of the 6508 total genes encoded by the *B. thailandensis* strain E555 genome, RNA-seq analysis detected expression of approximately 88% of the genes during growth in broth or in macrophages. In parallel mass spectrometry identified 42% of the predicted proteins. Similar findings have been reported in other bacteria [54–57]. Our finding that the transcriptome and proteome did not precisely mirror each other is not unexpected. In an *Escherichia coli* single-cell study [57] the correlation coefficient between mRNA and protein levels of the same gene averaged zero for the genes tested. Although according to the central dogma of molecular biology that transcription and translation are tightly linked in prokaryotes [58], the lack of correlation between mRNA and the encoded protein has been attributed to differences in the stability of these molecules [57] and in translation efficiency. Translation efficiency has a large impact on transcript-protein correlation. For example transcripts that have weak Shine-Dalgarno sequences are translated less efficiently [59, 60]. Secondary structure of the mRNA [61] and codon bias (large codon bias correlates with highly expressed genes [62, 63] and proteins [64]) also have an influence on translation efficiency. Untranslated RNA molecules are also considered to be responsible for the differences between the transcriptomic and the proteomic results. However, the most important factor responsible for low correlations between mRNA and protein expressions is the long half-life of proteins relative to mRNAs. Transcripts are instantaneous messengers that are degraded in 3 to 8 min [65], while proteins are accumulated products with a typical half-life of ∼20 h in *E. coli* [66, 67]. Finally, the reduced ability to recover some membrane-located or secreted proteins would limit the ability to detect them. These differences found between the transcriptomic and the proteomic data highlight the value of profiling both the transcriptome and the proteome.

Our comparison of the transcriptomes and proteomes of *B. thailandensis* strain E555 showed that most of the detected proteins were also present in the corresponding transcriptomes. We looked for transcripts and proteins that are significantly up-regulated during infection and found that an analysis of the transcriptome revealed more virulence-associated factors than an analysis of the proteome. However, although the proteome data revealed fewer virulence factors, they were identified with a higher degree of confidence. We found some examples of opposite patterns of gene and protein expression after infection (Additional file 7: Table S5). This raises the question of which method to use identifying candidate virulence factors. Among these genes we detected 1 virulence-associated factor (Fig|869,728.6.peg.5292) which was significantly up-regulated in the transcriptome but down-regulated in the proteome.

Both analyses generated false negative results. For example, deletion of the *treA* gene (BPSS0671) results in reduced intracellular survival of *B. pseudomallei* [68] but, compared to broth grown bacteria expression of the trehalase A gene in *B. thailandensis* strain E555 (Fig|869,728.6.peg.4715) was reduced during macrophage infection and the protein was not detected in culture or in macrophages. We also found numbers of other known virulence factors in the significantly down-regulated groups (Additional file 7: Table S5). This may reflect the different roles of virulence factors in different cell types or different hosts or at different stages of infection.

In summary, we combined a transcriptomic and proteomic approach to elucidate the changes involved in the adaptation of *B. thailandensis* strain E555 to macrophage cells. The differences found between the transcriptomic and the proteomic data suggested various post-transcriptional mechanisms and the complexity of the bacterial adaptation.

## Conclusions

In contrast with *B. thailandensis* strain E264, which has been used widely as a surrogate model for *B. pseudomallei*, we show that the pattern of growth of *B. thailandensis* strain E555 in macrophages more closely mirrors that of *B. pseudomallei*. Using this infection model we have shown that many of the known virulence factors of *B. pseudomallei* can be identified as genes or proteins whose expression is elevated on the infection of macrophages. This finding further confirms the utility of *B. thailandensis* strain E555 as a surrogate for *B. pseudomallei*. The identification of up-regulated genes provided a more comprehensive identification of virulence factors than the identification of up-regulated proteins.

## Methods

### Bacterial strain and cell culture growth conditions

*B. thailandensis* strain E555 (gift from Patrick Tan, Genome Institute of Singapore) was grown on Luria-Bertani (LB) agar or in LB broth (200 rpm) at 37 °C. J774A.1 mouse macrophage cells (European Collection of Cell Cultures, ECACC; Catalog No. 91051511) were maintained at 37 °C under 5% CO_2_ atmosphere in DMEM (with 4.5 g/l Glucose, 4.0 mM L-Glutamine and Sodium Pyruvate; Gibco 11,995,073) supplemented with 10% heat inactivated fetal bovine serum (FBS Gold; PAA A15–751).

### Macrophage infection model

For infection assays, 1.5 × 10^5^ cells/well in 24-well cell culture plates or 6 × 10^6^ cells/T75 flask (approximately 7.9 × 10^4^ cells/cm^2^) were seeded and an overnight culture of bacteria was diluted to 1.5 × 10^6^ cells/ml or to 6 × 10^7^ cells/10 ml in Leibovitz L-15 Media (with 2.05 mM L-Glutamine; Gibco 21,083,027), respectively. J774A.1 monolayers were washed once with L-15 medium before addition of bacteria at a multiplicity of infection of 10 (1 ml/well or 10 ml/T75 flask). Murine macrophages with the bacterial cells were incubated at 37 °C for 2 h to allow bacterial internalisation to occur. Extracellular bacteria were removed by washing the macrophage cells three times with warm PBS (Gibco 10,010,023). Fresh media containing kanamycin (1 mg/ml, which was replaced with 0.25 mg/ml kanamycin after 2 h of incubation) was then added to each well or flask to suppress the growth of extracellular bacteria. Infected cells were incubated in the presence of kanamycin at 37 °C until washed three times with PBS at appropriate time points. To determine the number of intracellular bacteria, macrophages were lysed in 0.1% Triton X-100 (Sigma-Aldrich T8787) in PBS for 5 mins, the lysis mixture was serially diluted and plated out on LB agar plates, which were then incubated overnight at 37 °C to allow bacteria to grow. Unless otherwise stated, all infections were performed as three independent experiments, each with three technical replicates. Macrophages infected in T75 flasks were processed for transcriptome and proteome analysis as detailed below.

### Genome sequencing and assembly

DNA were prepared for sequencing using a Promega Wizzard Genomic DNA Purification kit according to the manufacturer’s instructions. Sequencing was performed at the University of Exeter Sequencing Facility using an ‘Illumina HiSeq 2500 System’ benchtop sequencing instrument (read length: 100 bp, read type: paired end). Illumina adapters were removed and sequences quality trimmed using ea-utils [69]. SPAdes [70] was used to perform a de-novo assembly of the samples. This Whole Genome Shotgun project has been deposited at DDBJ/ENA/GenBank under the accession SJET00000000.

### Bacterial RNA extraction, sequencing and reverse transcription quantitative real-time PCR (RT-qPCR) for transcriptional studies

Total RNA was extracted from infected macrophages (internalised CFU per replicate approximately 1.5 × 10^7^ and 2 × 10^7^ at 5 and 6 h, respectively) and from bacterial cultures, and the RNA integrity number (RIN) was determined. RIN was more than 9 with all the extracts indicating no evidence of RNA degradation. After the final washing step at 5 or 6-h post-infection (2-h infection + 3 or 4-h kanamycin treatment), 2 ml TRIzol® Reagent (Invitrogen 15,596,026) was added to the flasks to lyse both the macrophage and bacterial cells. Cell lysates were stored at − 80 °C until further processing. When thawed, total RNA (total eukaryotic and total prokaryotic RNA) was extracted using the Direct-zol™ RNA MiniPrep Kit (Zymo Research R2052). Contaminating DNA was digested by DNAse I treatment (Ambion AM2222) which was confirmed by reverse transcriptase PCR (Qiagen OneStep RT-PCR Kit 210,212) using primers *glt1*-F (5` CGCACCATGACATCTATTCG 3`) vs *glt1*-R (5` ACCGGATTGACGTTCTTCAG 3`). DNA-free RNA was treated with the TruSeq RNA Library Preparation Kit v2 (Illumina RS-122-2001) and the Ribo-Zero Gold rRNA Removal Kit (Epidemiology) (Illumina MRZE724). Eukaryotic mRNA was first removed using poly-A selection, the remaining RNA was then enriched for bacterial mRNA by depleting both the bacterial and macrophage rRNA and the RNA-seq libraries were prepared to the guidelines of the manufacturer. Control bacterial mRNA was obtained from the overnight broth cultures of bacteria which were used to infect macrophage cells. RNA from broth culture was isolated as described above (including the poly-A depletion step). RNA was isolated from 3 separate assays (biological replicates) from both infected macrophages and control bacteria. RNA-seq libraries were created using the Illumina TruSeq Stranded mRNA Library Prep Kit according to the manufacturer’s protocol. The concentration, quality and integrity of all RNA and DNA samples were analysed using the Agilent 2100 Bioanalyser. Sequencing was performed at the University of Exeter Sequencing Facility using an ‘Illumina MiSeq System’ benchtop sequencing instrument (read length: 75 bp, read type: paired end). Reads from RNASeq were mapped to the *B. thailandensis* strain E555 genome assembly using Tophat [71]. Cufflinks [72] was used for transcript assembly of individual samples. All assemblies were merged to create a reference transcript, which was used to calculate gene counts with HTSeq [73]. DESeq [74] was then used to find differentially expressed genes. Transcripts with a *p*-value< 0.05 and more than 2-fold differential expression were considered significantly expressed.

The preparation of cDNA from total RNA extracted from culture and infected macrophages was carried out using the Superscript III Reverse Transcriptase Synthesis System (Invitrogen 18,080,051) with random hexamers according to the manufacturer’s recommendations. RT-qPCR was performed with primers annealing to internal regions of the target genes (Additional file 10: Table S8) using Platinum® SYBR® Green qPCR SuperMix-UDG according to the manufacturer’s manual (Invitrogen 11,733,038). For the adjustment of cDNA amounts, the housekeeping gene 23S rRNA [75] was used as internal standard.

### Bacterial protein extraction and mass spectrometric analysis for proteomic studies

In contrast to the RNA, prokaryotic and eukaryotic proteins are indistinguishable. Therefore, intact bacterial cells were first extracted from infected macrophages and bacterial proteins were then isolated from the bacterial cells. Macrophages were infected as described above using the same conditions as used for the 6 h transcriptome samples (i.e. internalised CFU per replicate approximately 2 × 10^7^ at 6 h post-infection). Following the final washing step at 6-h post-infection (2-h infection + 4-h kanamycin treatment) infected macrophages in T75 flasks were lysed in 0.1% Triton X-100 in PBS for 5 mins to release the intracellular bacteria and whole bacterial cells were isolated using a modified immunomagnetic separation technique described previously by Twine et al. [76]. Briefly, Dynabeads® M-270 Epoxy (Life Technologies 14311D) were covalently attached to goat α-mouse IgG (H + L) secondary antibodies (Invitrogen A16068) as per the manufacturer’s instructions (20 μg of antibodies per 1 mg of magnetic beads were used) and magnetic beads coated with secondary antibodies were labelled with mouse α-capsule primary antibodies (CPS 4V1H12 [12]) (17.5 μg of α-CPS antibodies per 1 mg of bead – secondary antibody complexes), which recognise surface antigens of *B. thailandensis* strain E555 cells. Magnetic bead – α-mouse – α-CPS complexes were then used to purify the bacteria. 1 mg of beads coated with both antibodies was mixed with macrophage cell lysate per T75 flask and incubated for 25 min with constant agitation (35 rpm) at 4 °C to capture the bacterial cells. The beads-bacteria complexes were separated from the cell debris on a magnetic stand, washed, and immediately re-suspended in BugBuster® Protein Extraction Reagent (Novagen 70,584) to lyse the bacterial cells and release the proteins. Bacterial purification was completed in about 45 min. Control extracts included bacterial proteins isolated by BugBuster® Protein Extraction Reagent from the overnight broth cultures of bacteria which were used to infect macrophage cells.

Mass spectrometric analysis of protein samples isolated from 3 biological replicates of both infected macrophages and bacterial cultures was done by the University of Bristol Proteomics Facility. Proteomics was performed as described previously using an UltiMate™ 3000 nano HPLC system in line with an LTQ-Orbitrap Velos mass spectrometer (Thermo Scientific) [77]. The raw data files were processed and quantified using Proteome Discoverer software v1.2 (Thermo Scientific) and searched against *B. thailandensis* strain E555 RAST ORFs using the SEQUEST algorithm. The reverse database search option was enabled and all peptide data was filtered to satisfy false discovery rate (FDR) of 5%. Abundance of each protein in each sample was calculated using the average area measurements of the three most abundant peptides matching to each protein (Top3 method) [78]. This value was then expressed in the fraction of the signal derived from the 100 most abundant proteins detected in each sample which was then compared for each protein in LB broth and macrophages. Statistical significance of the fold change difference was calculated using R with q-values reported after 5% FDR correction. All proteins with a q-value< 0.05 and more than 2-fold change difference were considered significantly regulated.

### Online and bioinformatic tools

Genome sequence of *B. thailandensis* strain E555 was annotated using the web-based RAST server (Rapid Annotation using Subsystem Technology) [44]. Cellular localisation of the predicted proteins encoded in the *B. thailandensis* strain E555 genome was predicted using PSORTb 3.0 [79] (Additional file 3: Table S1.D). Protein homologs in *B. pseudomallei* strain K96243, *B. thailandensis* strain E555 and *B. thailandensis* strain E264 were identified by blast search. Proteins were considered homologs if they met the following criteria: e value ≤0.000001 and (i) sequence identity > 30% if alignment length/query length > 90% [80], (ii) sequence identity > 40% if alignment length/query length between 70 and 90%, (iii) sequence identity > 55% if alignment length/query length between 50 and 70% (Additional file 3: Table S1.C). Correlation coefficients between the samples were calculated using the formula built-in the Microsoft Excel Software. *B. thailandensis* strain E555 genes were classified into functional categories based on clusters of orthologous gene (COG) designations; functional classes of *B. thailandensis* genes and proteins were determined based on the categorisation of their homologs in *B. pseudomallei* (Sheet C of Additional file 4: Table S2 in reference [81], Additional file 3: Table S1 D). Statistical analysis of growth curves was performed using Graph Pad Prism 5.03 software One-way ANOVA Kruskal-Wallis test with Dunns post test. DESeq [74] was used to determine fold change and the *p* value of differential expression in the RNASeq data. Statistical significance (*q* value) of the fold change difference in the proteome was calculated using R. To identify pathways that are up- or down-regulated during infection we used Keg Array 1.2.4a software which maps the genes/proteins to KEGG pathways. Up-regulation or down-regulation of genes/proteins with at least 1.5-fold increase or decrease in expression difference was considered to be necessary or dispensable respectively during macrophage infection.

## Additional file

Additional file 1: **Figure S1.** Abundance of transcripts detected in the bacterial transcriptome both at 5 h and 6 h post infection. **Figure S2.** Abundance of bacterial transcripts detected in culture and in macrophages. **Figure S3.** Abundance of bacterial proteins detected in culture and in macrophages. **Figure S4.** Volcano plots of gene expression for *B. thailandensis* strain E555 during macrophage infection (versus *in vitro* growth) at transcript and protein level. (DOCX 1997 kb)

Additional file 2: **Supplementary References.** (DOCX 17 kb)

Additional file 3: **Table S1.** Details of *B. thailandensis* strain E555 genes and proteins. (XLSX 6051 kb)

Additional file 4: **Table S2.** Gene expression results and details of the genes subjected to RT-qPCR analysis. (XLSX 15 kb)

Additional file 5: **Table S3.** Transcripts detected in vitro and in vivo. (XLSX 2144 kb)

Additional file 6: **Table S4.** Proteins detected in vitro and in vivo. (XLSX 1095 kb)

Additional file 7: **Table S5.** Significantly regulated genes, capsule expression and all expression data. (XLSX 2279 kb)

Additional file 8: **Table S6.** Known virulence genes. (XLSX 208 kb)

Additional file 9: **Table S7.** Lists of genes in each group shown in Table 2. (XLSX 321 kb)

Additional file 10: **Table S8.** Primers used in RT-qPCR. (DOCX 15 kb)

## Abbreviations

CFU: Colony forming unit
CFU: Colony-forming unit
COG: Clusters of orthologous gene
CPS: Capsule polysaccharide
DMEM: Dulbecco’s modified Eagle’s medium
DNA: Deoxyribose nucleic acid
LB: Luria-Bertani
mRNA: Messenger ribonucleic acid
RAST: Rapid Annotation using Subsystem Technology
RNA-Seq: RNA sequencing
RT-qPCR: Reverse transcription quantitative real-time polymerase chain reaction
SDS-PAGE: Sodium dodecyl sulfate polyacrylamide gel electrophoresis
T3SS: Type III secretion system
T6SS: Type VI secretion system
