# Supplementary material for: Burkholderia thailandensis strain E555 is a surrogate for the investigation of Burkholderia pseudomallei replication and survival in macrophages
Source: BMC Microbiol. 2019 May 15;19:97. doi: 10.1186/s12866-019-1469-8 (PMC6521459; doi:10.1186/s12866-019-1469-8)
Supplement: Supplementary file 1 — Figure S1. Abundance of transcripts detected in the bacterial transcriptome both at 5 h and 6 h post infection. Figure S2. Abundance of bacterial transcripts detected in culture and in macrophages. Figure S3. Abundance of bacterial proteins detected in culture and in macrophages. Figure S4. Volcano plots of gene expression for B. thailandensis strain E555 during macrophage infection (versus in vitro growth) at transcript and protein level. (DOCX 1997 kb) [file 12866_2019_1469_MOESM1_ESM.docx]

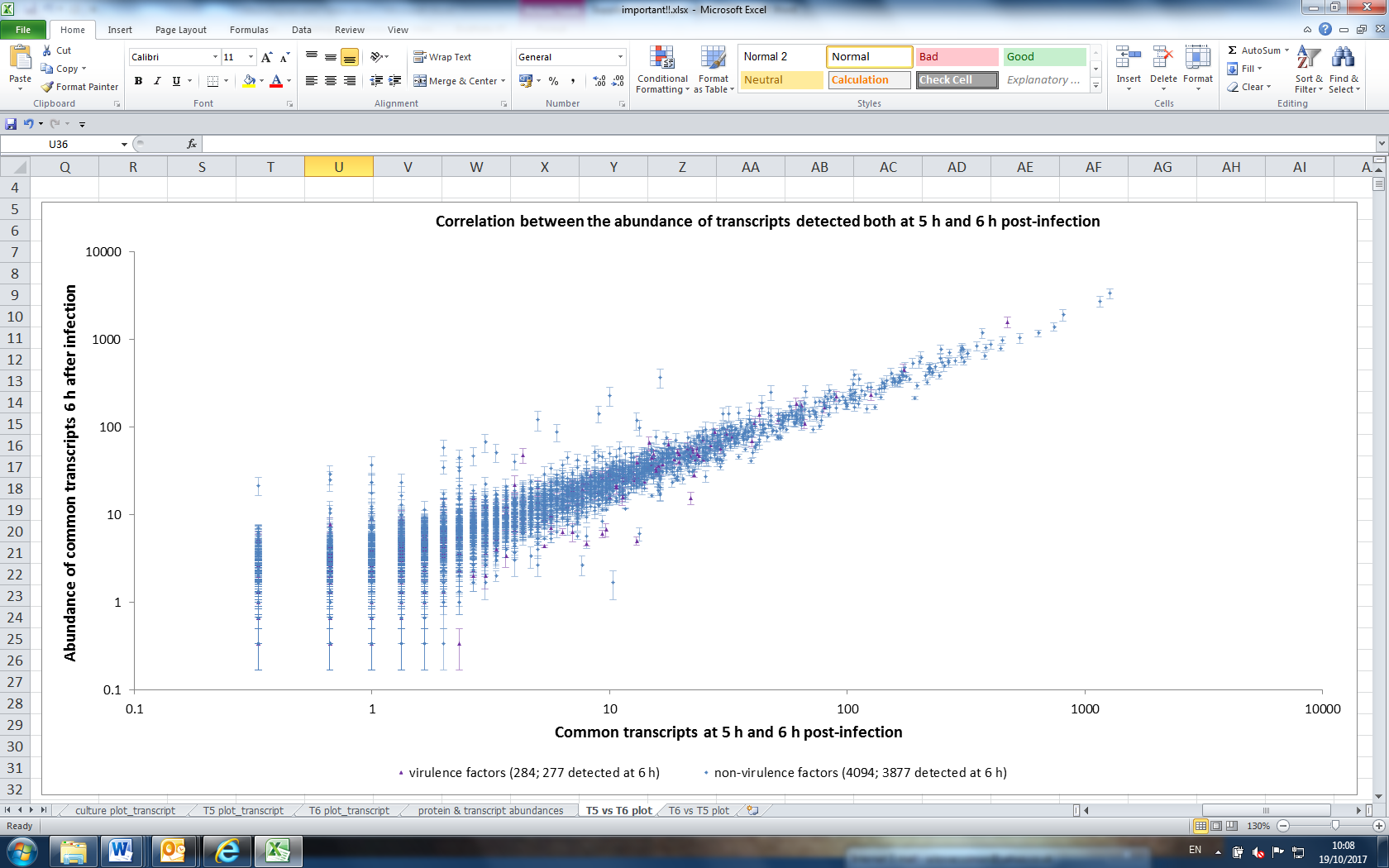


**A**

**B**


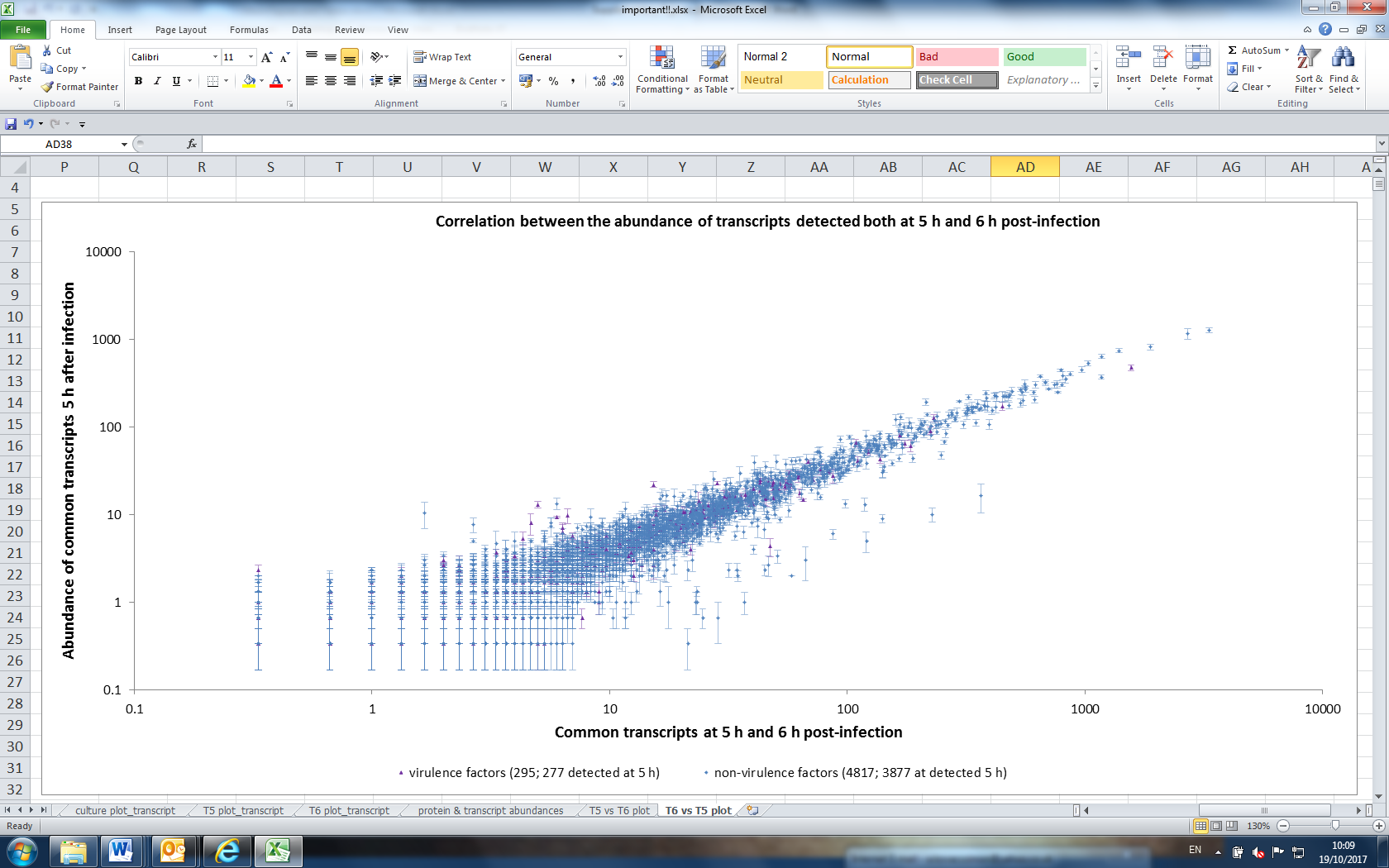


**Supplementary Figure S1.** **Abundance of transcripts detected in the bacterial transcriptome both at 5 h and 6 h post-infection.** (A) Abundance of common transcripts at 6 h post-infection, ordered by increasing abundance at 5 h post-infection. (B) Abundance of common transcripts at 5 h post-infection, ordered by increasing abundance at 6 h post-infection.


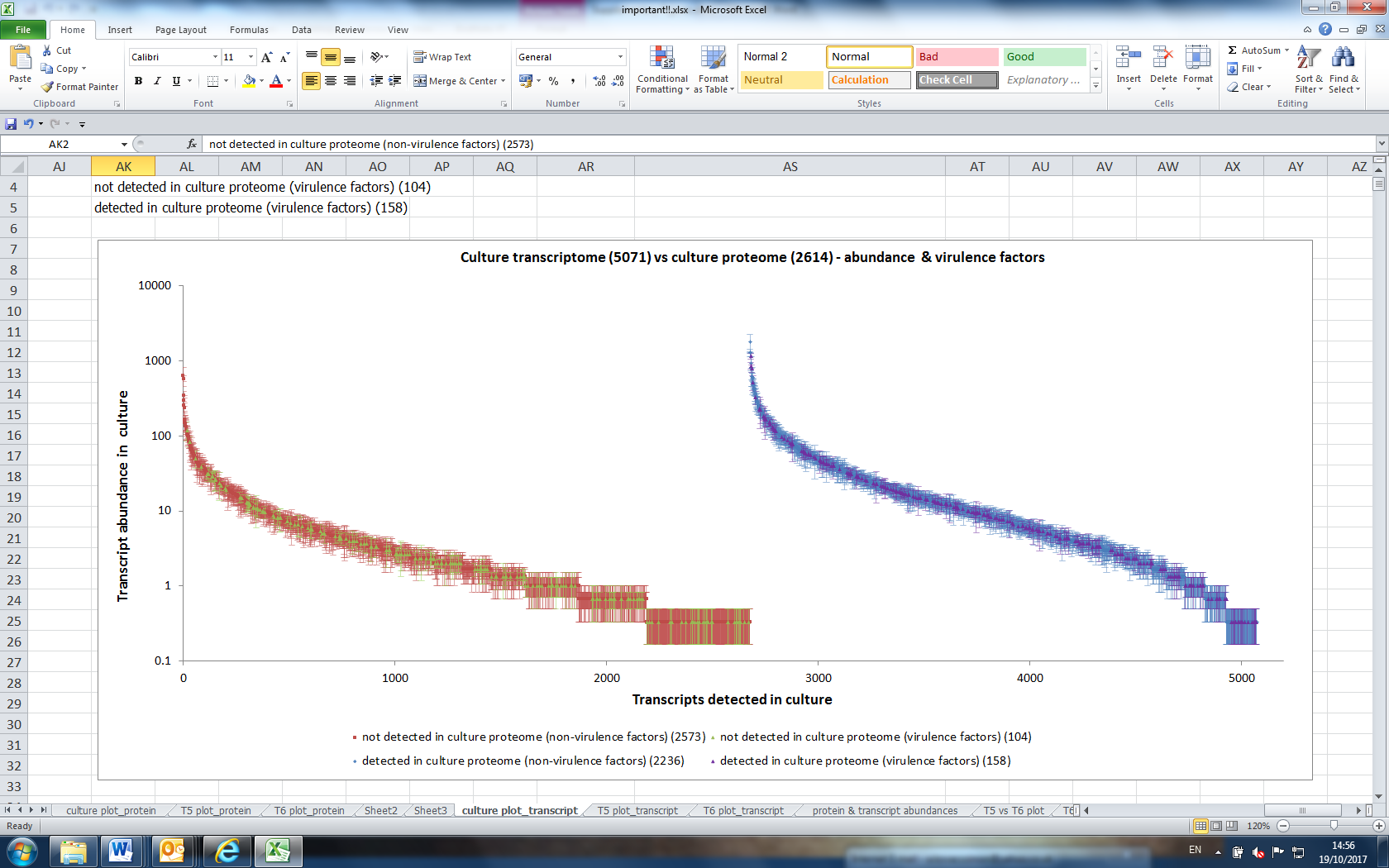


**A**

**B**


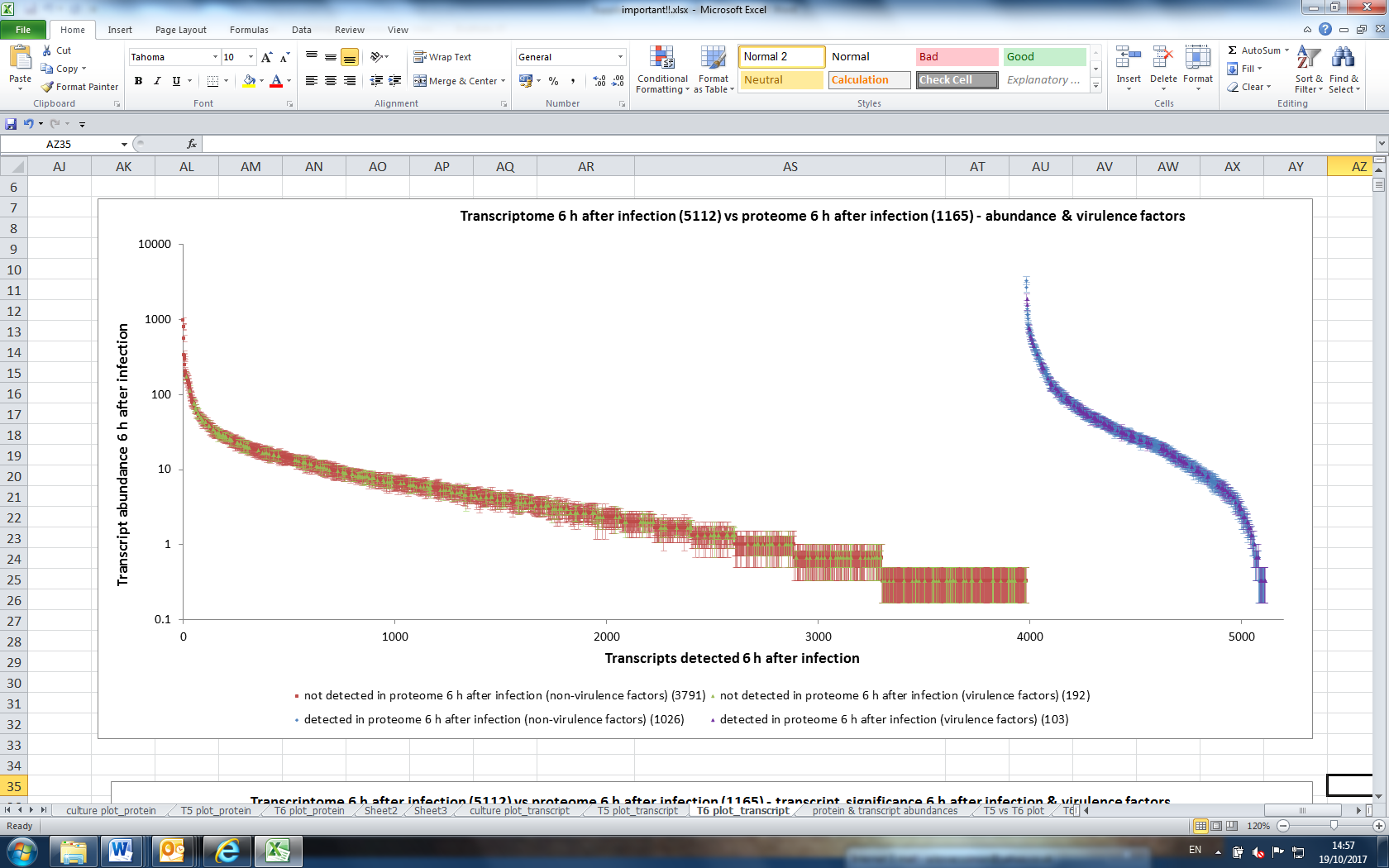


**Supplementary Figure S2. Abundance of bacterial transcripts detected in culture and in macrophages.** Transcripts on the horizontal axes are listed in two groups; transcripts shown on the left were not detected in the corresponding proteome (brown and green), while transcripts shown on the right were present in the corresponding proteome (purple and blue). (A) Abundance of transcripts detected in culture. (B) Abundance of transcripts detected in macrophages 6 h after infection.


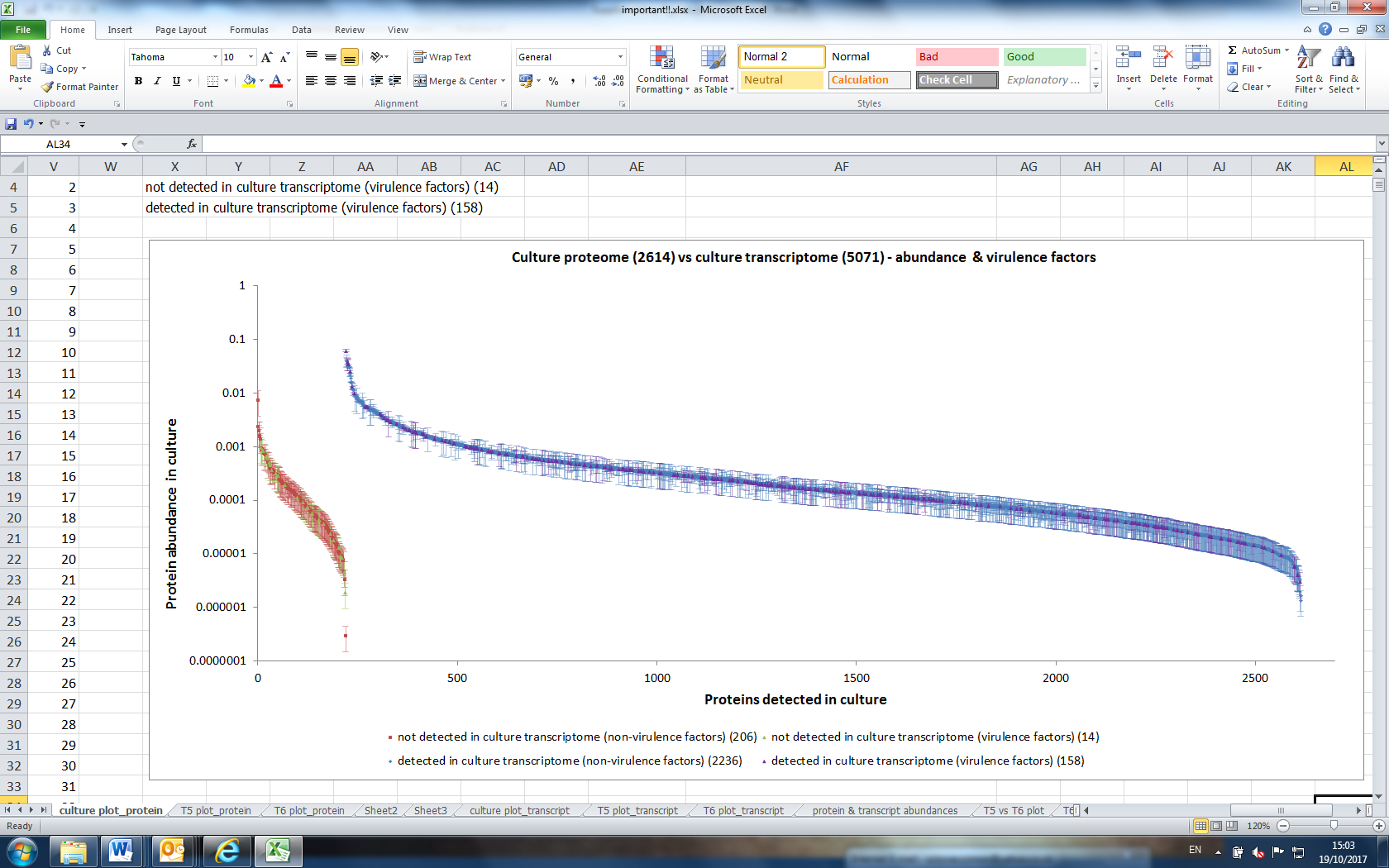


**A**

**C**


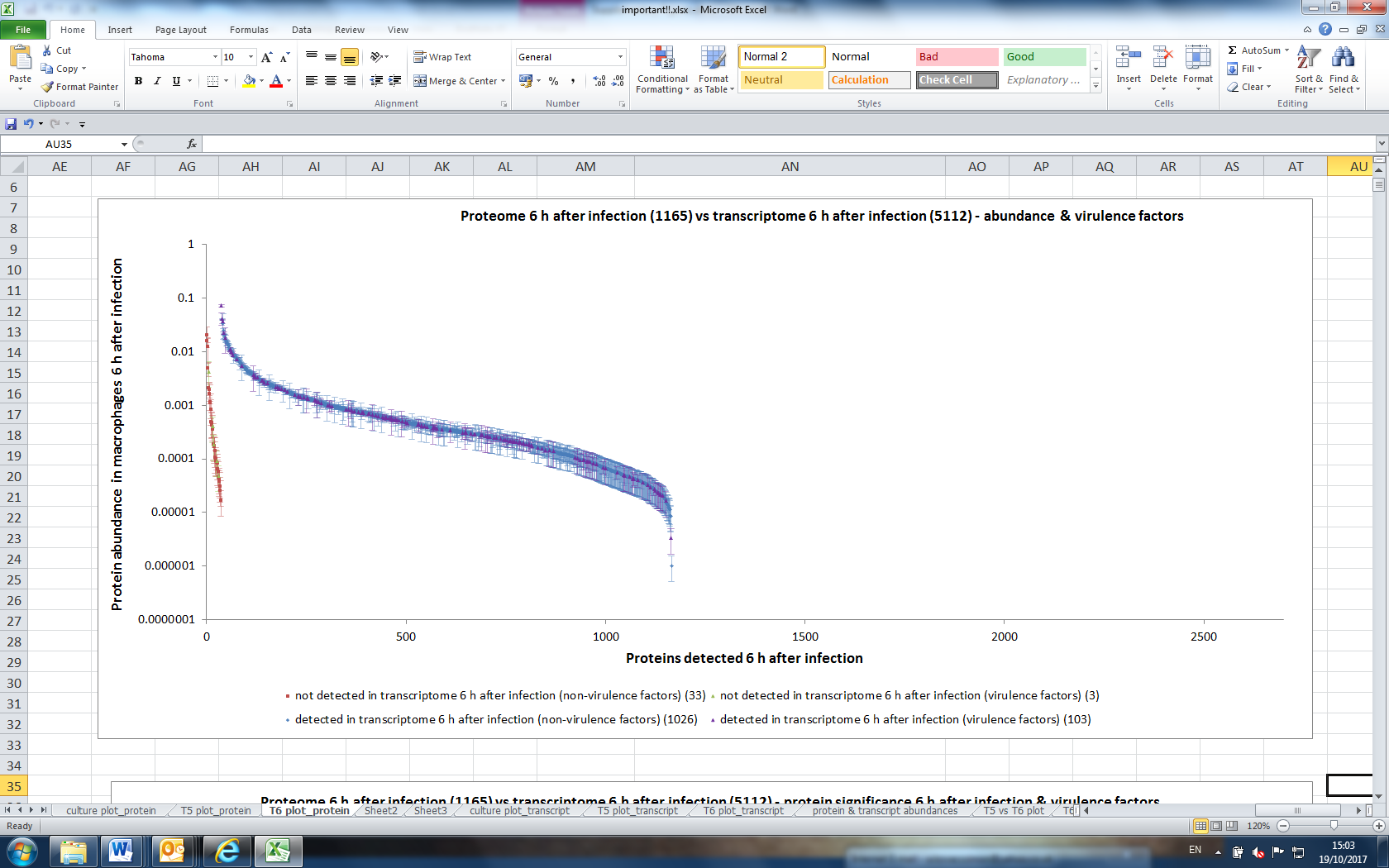


**Supplementary Figure S3. Abundance of bacterial proteins detected in culture and in macrophages.** Proteins on the horizontal axes are listed in two groups; proteins shown on the left were not detected in the corresponding transcriptome (brown and green), while proteins shown on the right were present in the corresponding transcriptome (purple and blue). (A) Abundance of proteins detected in culture. (B) Abundance of proteins detected in macrophages 6 h after infection, versus transcripts detected in macrophages 6 h after infection.

**A**


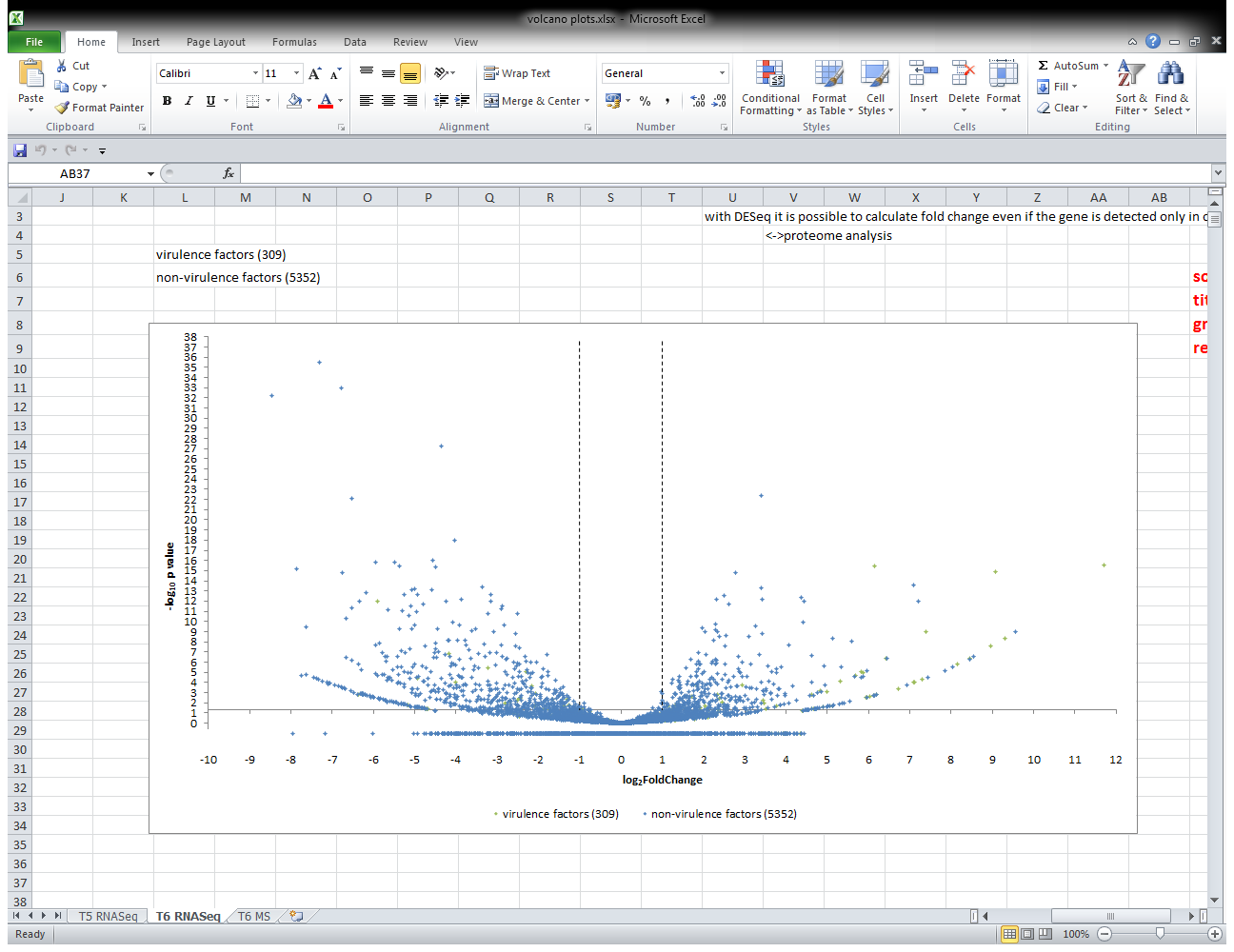


**B**


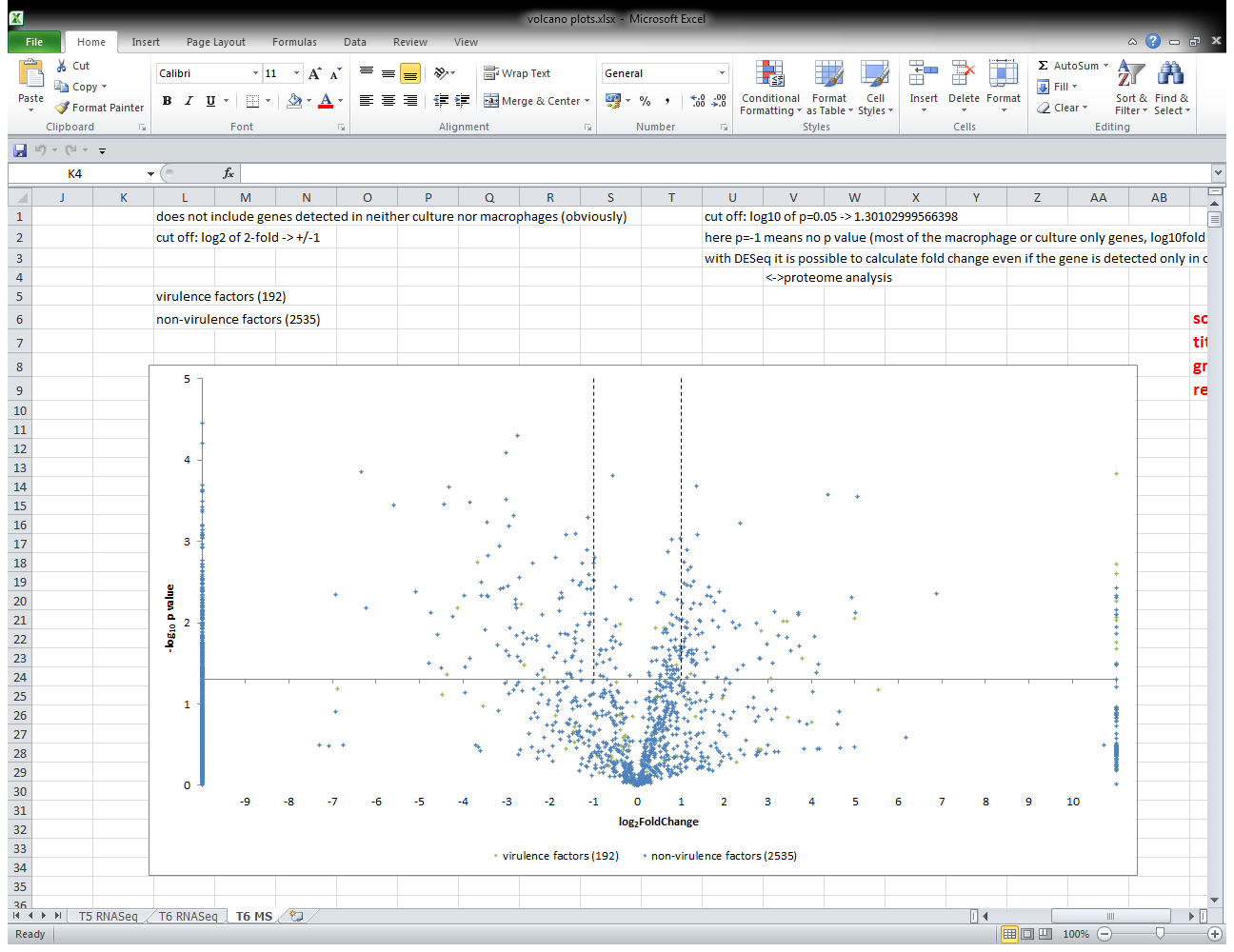


**Supplementary Figure S4. Volcano plots of gene expression for *B. thailandensis* strain E555 during macrophage infection (versus *in vitro* growth) at transcript and protein level.** Gated transcripts and proteins correspond to -1 > log2 > 1 differential expression and p < 0.05 (-log_10_p > 1.30102999566398) significance. (A) Distribution of differentially expressed genes at 6 h post-infection. (B) Distribution of differentially expressed proteins at 6 h post-infection.
