# Supplementary material for: Burkholderia thailandensis strain E555 is a surrogate for the investigation of Burkholderia pseudomallei replication and survival in macrophages
Source: BMC Microbiol. 2019 May 15;19:97. doi: 10.1186/s12866-019-1469-8 (PMC6521459; doi:10.1186/s12866-019-1469-8)
Supplement: Supplementary file 2 — Supplementary References. (DOCX 17 kb) [file 12866_2019_1469_MOESM2_ESM.docx]

**Supplementary References**

1. Atkins, T., et al., *A mutant of Burkholderia pseudomallei, auxotrophic in the branched chain amino acid biosynthetic pathway, is attenuated and protective in a murine model of melioidosis.* Infect Immun, 2002. **70**(9): p. 5290-4.

2. Breitbach, K., J. Kohler, and I. Steinmetz, *Induction of protective immunity against Burkholderia pseudomallei using attenuated mutants with defects in the intracellular life cycle.* Trans R Soc Trop Med Hyg, 2008. **102 Suppl 1**: p. S89-94.

3. Burtnick, M.N., et al., *Burkholderia pseudomallei type III secretion system mutants exhibit delayed vacuolar escape phenotypes in RAW 264.7 murine macrophages.* Infect Immun, 2008. **76**(7): p. 2991-3000.

4. Campos, C.G., M.S. Byrd, and P.A. Cotter, *Functional characterization of Burkholderia pseudomallei trimeric autotransporters.* Infect Immun, 2013. **81**(8): p. 2788-99.

5. Champion, O.L., et al., *Immunisation with proteins expressed during chronic murine melioidosis provides enhanced protection against disease.* Vaccine, 2016. **34**(14): p. 1665-71.

6. Chen, Y., et al., *Characterization and analysis of the Burkholderia pseudomallei BsaN virulence regulon.* BMC Microbiol, 2014. **14**: p. 206.

7. Chirakul, S., et al., *Characterization of BPSS1521 (bprD), a regulator of Burkholderia pseudomallei virulence gene expression in the mouse model.* PLoS One, 2014. **9**(8): p. e104313.

8. Chua, K.L., Y.Y. Chan, and Y.H. Gan, *Flagella are virulence determinants of Burkholderia pseudomallei.* Infect Immun, 2003. **71**(4): p. 1622-9.

9. Cuccui, J., et al., *Development of signature-tagged mutagenesis in Burkholderia pseudomallei to identify genes important in survival and pathogenesis.* Infect Immun, 2007. **75**(3): p. 1186-95.

10. Cummings, J.E., et al., *The Burkholderia pseudomallei enoyl-acyl carrier protein reductase FabI1 is essential for in vivo growth and is the target of a novel chemotherapeutic with efficacy.* Antimicrob Agents Chemother, 2014. **58**(2): p. 931-5.

11. Erskine, P.T., et al., *High resolution structure of BipD: an invasion protein associated with the type III secretion system of Burkholderia pseudomallei.* J Mol Biol, 2006. **363**(1): p. 125-36.

12. Gong, L., et al., *The Burkholderia pseudomallei type III secretion system and BopA are required for evasion of LC3-associated phagocytosis.* PLoS One, 2011. **6**(3): p. e17852.

13. Gutierrez, M.G. and J.M. Warawa, *Attenuation of a select agent-excluded Burkholderia pseudomallei capsule mutant in hamsters.* Acta Trop, 2016. **157**: p. 68-72.

14. Hasselbring, B.M., M.K. Patel, and M.A. Schell, *Dictyostelium discoideum as a model system for identification of Burkholderia pseudomallei virulence factors.* Infect Immun, 2011. **79**(5): p. 2079-88.

15. Hopf, V., et al., *BPSS1504, a cluster 1 type VI secretion gene, is involved in intracellular survival and virulence of Burkholderia pseudomallei.* Infect Immun, 2014. **82**(5): p. 2006-15.

16. Ireland, P.M., et al., *Disarming Burkholderia pseudomallei: structural and functional characterization of a disulfide oxidoreductase (DsbA) required for virulence in vivo.* Antioxid Redox Signal, 2014. **20**(4): p. 606-17.

17. Lazar Adler, N.R., et al., *Systematic mutagenesis of genes encoding predicted autotransported proteins of Burkholderia pseudomallei identifies factors mediating virulence in mice, net intracellular replication and a novel protein conferring serum resistance.* PLoS One, 2015. **10**(4): p. e0121271.

18. Lazar Adler, N.R., et al., *Perturbation of the two-component signal transduction system, BprRS, results in attenuated virulence and motility defects in Burkholderia pseudomallei.* BMC Genomics, 2016. **17**(1): p. 331.

19. Lee, H.S., et al., *CdpA is a Burkholderia pseudomallei cyclic di-GMP phosphodiesterase involved in autoaggregation, flagellum synthesis, motility, biofilm formation, cell invasion, and cytotoxicity.* Infect Immun, 2010. **78**(5): p. 1832-40.

20. Moule, M.G., et al., *Characterization of New Virulence Factors Involved in the Intracellular Growth and Survival of Burkholderia pseudomallei.* Infect Immun, 2016. **84**(3): p. 701-10.

21. Muangsombut, V., et al., *Inactivation of Burkholderia pseudomallei bsaQ results in decreased invasion efficiency and delayed escape of bacteria from endocytic vesicles.* Arch Microbiol, 2008. **190**(6): p. 623-31.

22. Muller, C.M., et al., *Role of RelA and SpoT in Burkholderia pseudomallei virulence and immunity.* Infect Immun, 2012. **80**(9): p. 3247-55.

23. Neamnak, J., et al., *Comparison of stress adaptation and survival rate between Burkholderia pseudomallei with mutant and wild type bfmR.* Southeast Asian J Trop Med Public Health, 2014. **45**(2): p. 346-51.

24. Norris, M.H., et al., *Glyphosate resistance as a novel select-agent-compliant, non-antibiotic-selectable marker in chromosomal mutagenesis of the essential genes asd and dapB of Burkholderia pseudomallei.* Appl Environ Microbiol, 2009. **75**(19): p. 6062-75.

25. Norris, M.H., et al., *The Burkholderia pseudomallei Deltaasd mutant exhibits attenuated intracellular infectivity and imparts protection against acute inhalation melioidosis in mice.* Infect Immun, 2011. **79**(10): p. 4010-8.

26. Norville, I.H., et al., *A novel FK-506-binding-like protein that lacks peptidyl-prolyl isomerase activity is involved in intracellular infection and in vivo virulence of Burkholderia pseudomallei.* Microbiology, 2011. **157**(Pt 9): p. 2629-38.

27. Ooi, W.F., et al., *The condition-dependent transcriptional landscape of Burkholderia pseudomallei.* PLoS Genet, 2013. **9**(9): p. e1003795.

28. Pilatz, S., et al., *Identification of Burkholderia pseudomallei genes required for the intracellular life cycle and in vivo virulence.* Infect Immun, 2006. **74**(6): p. 3576-86.

29. Reckseidler, S.L., et al., *Detection of bacterial virulence genes by subtractive hybridization: identification of capsular polysaccharide of Burkholderia pseudomallei as a major virulence determinant.* Infect Immun, 2001. **69**(1): p. 34-44.

30. Singh, A.P., et al., *Evolutionary analysis of Burkholderia pseudomallei identifies putative novel virulence genes, including a microbial regulator of host cell autophagy.* J Bacteriol, 2013. **195**(24): p. 5487-98.

31. Srilunchang, T., et al., *Construction and characterization of an unmarked aroC deletion mutant of Burkholderia pseudomallei strain A2.* Southeast Asian J Trop Med Public Health, 2009. **40**(1): p. 123-30.

32. Stevens, M.P., et al., *A Burkholderia pseudomallei type III secreted protein, BopE, facilitates bacterial invasion of epithelial cells and exhibits guanine nucleotide exchange factor activity.* J Bacteriol, 2003. **185**(16): p. 4992-6.

33. Stone, J.K., et al., *Melioidosis: molecular aspects of pathogenesis.* Expert Rev Anti Infect Ther, 2014. **12**(12): p. 1487-99.

34. Tunpiboonsak, S., et al., *Role of a Burkholderia pseudomallei polyphosphate kinase in an oxidative stress response, motilities, and biofilm formation.* J Microbiol, 2010. **48**(1): p. 63-70.

35. Vanaporn, M., et al., *Superoxide dismutase C is required for intracellular survival and virulence of Burkholderia pseudomallei.* Microbiology, 2011. **157**(Pt 8): p. 2392-400.

36. Wagley, S., et al., *The twin arginine translocation system is essential for aerobic growth and full virulence of Burkholderia thailandensis.* J Bacteriol, 2014. **196**(2): p. 407-16.

37. Yam, H., et al., *The multiple roles of hypothetical gene BPSS1356 in Burkholderia pseudomallei.* PLoS One, 2014. **9**(6): p. e99218.

38. Zajdowicz, S.L., et al., *Alanine racemase mutants of Burkholderia pseudomallei and Burkholderia mallei and use of alanine racemase as a non-antibiotic-based selectable marker.* PLoS One, 2011. **6**(6): p. e21523.
