# Supplementary material for: Burkholderia thailandensis strain E555 is a surrogate for the investigation of Burkholderia pseudomallei replication and survival in macrophages
Source: BMC Microbiol. 2019 May 15;19:97. doi: 10.1186/s12866-019-1469-8 (PMC6521459; doi:10.1186/s12866-019-1469-8)
Supplement: Supplementary file 10 — Table S8. Primers used in RT-qPCR. (DOCX 15 kb) [file 12866_2019_1469_MOESM10_ESM.docx]

**Supplementary Table S8.** Primers used in RT-qPCR.

| **Gene**  **Fig\|869728.6.** | **Primer name** | **Primer sequence (5`->3`)** | **Description/**  **Bp K96243 homolog** |
| --- | --- | --- | --- |
| **23S** | 23S-RT-F | GTAACGGAGGAGTACGAAGG | Housekeeping gene  BPSSr02, BPSLr02, BPSLr05, BPSLr08 |
|  | 23S-RT-R | GCGGTATCAGCCTGTTATCC |  |
| **peg.1728** | peg.1728-RT-F | GAAACGACACGCGATTCCAG | BPSS1729 |
|  | peg.1728-RT-R | CAACTCGCGCAGTTCAGTG |  |
| **peg.355** | peg.355-RT-F | GATCAAGACGCGCTTCGAC | BPSL0856 |
|  | peg.355-RT-R | GCTCGAACACCGAAATCATC |  |
| **peg.5948** | peg.5948-RT-F | GAGCACGTGACGATCTGGAG | *bsaR* (BPSS1542) |
|  | peg.5948-RT-R | AGGTCCGCGTAGAACGTCTC |  |
| **peg.731** | peg.731-RT-F | GATCCCGGTCGAGATGTTC | *tig* (BPSL1402) |
|  | peg.731-RT-R | GAATAATACCAGCGGACCAC |  |
| **peg.5909** | peg.5909-RT-F | GGGCAAGATTCTCGTCCAG | BPSS1498 |
|  | peg.5909-RT-R | CAGAATCCGGTAGAACTCG |  |
| **peg.6274** | peg.6274-RT-F | CCGAGATCATGCTGCTGTTC | BPSS0359 |
|  | peg.6274-RT-R | GATTCGTCAGCGACGGATG |  |
| **peg.1473** | peg.1473-RT-F | TTCGACGCGTTCAAGGAAG | *sodB* (BPSL0880) |
|  | peg.1473-RT-R | GCGTTGCGGTAATCGATG |  |
| **peg.4093** | peg.4093-RT-F | GCAGCTCGTCAACTCCAAG | *flgK* (BPSL0280) |
|  | peg.4093-RT-R | GCTTCCTCGTTCTGGTTCAC |  |
| **peg.5186** | peg.5186-RT-F | GAAGAGAAGCTCGCTTCGATG | BPSS2288 |
|  | peg.5186-RT-R | GCTCGCCTTCCTTCAGTTC |  |
